# A Comprehensive Review of In Situ Measurement Techniques for Evaluating the Electro-Chemo-Mechanical Behaviors of Battery Electrodes

**DOI:** 10.3390/molecules29081873

**Published:** 2024-04-19

**Authors:** Hainan Jiang, Jie Chen, Xiaolin Li, Zhiyao Jin, Tianjun Chen, Jiahui Liu, Dawei Li

**Affiliations:** 1 School of Mechanical Engineering, University of Shanghai for Science and Technology, Shanghai 200093, China; 18521511206@163.com (H.J.); lxl91331123@163.com (X.L.); jinzhiyao_123@163.com (Z.J.); liujiahui_116@163.com (J.L.); 2 Office of Research Affairs, Shanghai Institute of Satelite Engineering, Shanghai 201109, China; cj11271127@163.com; 3 State Key Laboratory of Space Power-Sources, Shanghai Institute of Space Power-Sources, Shanghai 200245, China; 13127921506@163.com

**Keywords:** multi-beam optical stress sensor, digital image correlation, bending curvature measurement system, lithium-ion batteries, measurement configurations, applications

## Abstract

The global production landscape exhibits a substantial need for efficient and clean energy. Enhancing and advancing energy storage systems are a crucial avenue to optimize energy utilization and mitigate costs. Lithium batteries are the most effective and impressive energy utilization system at present, with good safety, high energy density, excellent cycle performance, and other advantages, occupying most of the market. However, due to the defects in the electrode material of the battery itself, the electrode will undergo the process of expansion, stress evolution, and electrode damage during electro-chemical cycling, which will degrade battery performance. Therefore, the detection of property changes in the electrode during electro-chemical cycling, such as the evolution of stress and the modulus change, are useful for preventing the degradation of lithium-ion batteries. This review presents a current overview of measurement systems applied to the performance detection of batteries’ electrodes, including the multi-beam optical stress sensor (MOSS) measurement system, the digital image correlation (DIC) measurement system, and the bending curvature measurement system (BCMS), which aims to highlight the measurement principles and advantages of the different systems, summarizes a part of the research methods by using each system, and discusses an effective way to improve the battery performance.

## 1. Introduction

The global economy’s rapid growth has sharply increased the demand for clean and sustainable energy, positioning advanced energy storage systems as a pivotal strategy for sustainable development [1,2,3,4,5]. An excellent energy storage system should possess high energy density, superior energy conversion efficiency, and tolerance to a wide range of operation conditions [6,7,8,9,10]. Lithium batteries are generally composed of a cathode, liquid electrolytes, and an anode. They make a significant impact in the traditional energy storage system [11,12,13]. Meanwhile, as an excellent energy storage system, lithium batteries have been widely used in multiple areas, such as energy storage devices in aerospace systems and electric vehicles, etc.

Although lithium-ion batteries have advanced significantly in recent decades, further enhancements are required to fulfill increasing demands. For example, the over-charge and over-discharge of the batteries, the unbalanced charging problem, the impact of ambient temperature on battery life, and the mechanisms of stress effects, etc. The performance degradation of the batteries is influenced by various complex factors, including the working conditions, the cell design, and the working environment [14]. Usually, these factors combine to contribute to the degradation of the battery’s capacity, lifetime, and performance [15,16,17,18]. Therefore, in the process of improving the lithium-ion battery performance, researchers proposed different types of battery models with different test environments, which are used to improve the efficiency of performance monitoring and data collection during the lithium battery cycling process [19,20].

Moreover, stress significantly impacts lithium batteries’ capacity and durability, a subject of extensive discussion. We have a deep understanding of electro-chemically induced-stress and its influence mechanism on the electrode, which is vital for the development of next-generation lithium batteries. The multi-beam optical stress sensor (MOSS) measurement system is a typical system for measuring the stress evolution of electrode materials. Utilizing the Stoney equation, the MOSS system measures stress changes in battery electrodes by tracking curvature and thickness variations throughout electro-chemical cycles [21,22]. Sethuraman et al. used the MOSS system to monitor the diffusion-induced stress of the composite silicon electrodes and suggested that plasticity of the electrode can induce irreversible shape changes in the particles [23]. Li et al. proposed a novel method to measure the wafer warpage origination and evolution of multi-layered polyimide/Cu composite film in situ using the MOSS measurement system [24]. As an important negative active material for batteries, the composite graphite electrode is widely used because of its considerable first coulomb efficiency, reversible capacity, and excellent cycle stability. The digital image correlation (DIC) measurement system is more suitable for deformation observation and data acquisition of the electrode at large magnifications during electro-chemical cycling [25,26,27], which uses a camera and zoom lens to capture the fluorescence speckle patterns on the surface of the electrode excited by laser. The light reflected by fluorescence is captured by the camera, and forms a fluorescence spot pattern. Through the obtained pattern, displacement and deformation of the electrode can be calculated. Jones et al. studied the electrode mechanics of composite graphite electrode, and demonstrated the strain evolution with the capacity [28]. Chen et al. examined the relationship between capacity and mechanical deformation state during the electro-chemical cycling by using the DIC measurement system [29]. Wang et al. proposed the strain and stress information about the free-stranding *MWCNTs/V*_2_*O*_2_ composite electrode by combining the DIC measurement system and electro-chemical-mechanical constitutive equations, which showed that the strain is tensile and the stress is compressive. However, the typical composite electrodes in commercial lithium-ion batteries have a relatively complex porous structure. It usually consists of the current collector, active particles, polymeric binders and so on. Thus, it is difficult to measure the mechanical response and stress evolution in real time during the electro-chemical cycles. The bending curvature measurement system (BCMS) is an effective way to measure the deformation change in the composite electrode during electro-chemical cycling. Combined with the mathematical model and the established cantilever beam-bending configuration, the performance parameters of the composite electrode, such as the induced stress and modulus can be obtained [30,31]. Xie et al. combined the stress models and in situ deformation measurements to calculate the stress in the electrodes, which showed that the silicon and carbon materials exhibit softening or stiffening during the cycling [32]. All these measurement systems are classic means for measuring the performance of batteries and provide effective data references for the study of electrode degradation to design better lithium batteries. Apart from the three typical real-time measurement methods, there are many other material-characterized methods to achieve the same purpose, such as the peeling test [33,34,35], scratch [36,37,38,39,40], in situ scanning electron microscope (SEM) [41,42,43,44,45,46], in situ optical microscopy [47,48,49,50], in situ atomic force microscopy (AFM) [51,52,53,54,55], and in situ transmission electron microscopy (TEM) [56,57,58,59,60,61,62]. The characteristic length scale, advantages, and disadvantages of various in situ characterization tools for battery electrodes are shown in Table 1.

This article introduces and overviews three cell measurement systems: the multi-beam optical stress sensor (MOSS), digital image correlation (DIC), and the bending curvature measurement system (BCMS). Their collective summary in one paper stems from their complementary capabilities in assessing the electro-chemo-mechanical behaviors of battery electrodes. Each method provides unique insights into different aspects of electrode behavior under operational conditions, contributing to a holistic understanding of electrode performance and degradation mechanisms. Not only are the equipment configuration and the test principles of each measurement system presented, but the measurement details of the different systems are also introduced and discussed from the traditional performance indicators as induced stress, expansion strain, and modulus to the novel research targets. By summarizing the similarities and differences of the three systems in the process of lithium battery performance testing, we discuss the effective ways to improve the cycle life and performance of lithium batteries and promote the development of the lithium battery industry, which has certain reference value.

## 2. Multi-Beam Optical Stress Sensor (MOSS)

### 2.1. Experimental Device and Mathematical Model

#### 2.1.1. The Experimental Device and Basic Theory of the MOSS

**Figure 1:**
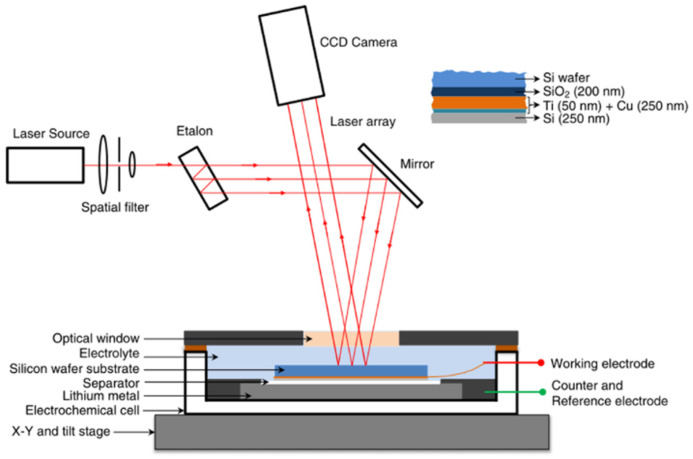
Illustration of the multi-beam optical stress sensor (MOSS) [54].

The MOSS has been proven to be an effective way to measure the coupling electro-mechanical properties of the electrode for lithium batteries [63]. Inside the electro-chemical cell, the Si film is the working electrode, and the Li metal is used as the counter and reference electrode, as shown in Figure 1. Meanwhile, the basic principle of this measurement system can be summarized as the variation in the laser beam spot spacing collected by the charge coupled device (CCD) camera during electro-chemical cycling, which is substituted into a mathematical model to obtain the corresponding signals of the electrode. Due to the large volume expansion of silicon electrodes during charge/discharge cycling, most researchers have used the MOSS to study silicon electrodes. Actually, the MOSS can also be applied to other materials such as graphite, LiCoO_2_, and so on.

#### 2.1.2. Mathematical Model for the MOSS

By monitoring the curvature changes in the substrate, the stress in the Si thin-film electrode can be measured [21,22]. The Stoney equation can be used to combine the biaxial film stress (σ) and the curvature change (Δκ),

(1) $$\sigma =\sigma^{r}+\frac{E_{s}h_{s}^{2}\Delta \kappa }{6h_{f}(1-\upsilon_{s})}$$

where $E_{s}$ and $\upsilon_{s}$ are the material parameters, while $h_{s}$ and $h_{f}$ are the structural parameters, respectively [64]. Based on detection of the height and volume changes [65], the film thickness during the lithiation/delithiation process can be assumed to be linear with SOC,

(2) $$h_{f}=h_{f}^{0}(1+2.7z)$$

where ${h_{f}}^{0}$ is the initial film thickness and *z* represents the SOC. The MOS technique employs an array of laser beams aligned in parallel, which are reflected from the surface of the sample and recorded by a camera, which is shown in Figure 1. The relationship between the change in spacing between the points of light and the curvature of the wafer is as follows:

(3) $$\kappa =\frac{\left(d-d^{0}\right)}{d^{0}}\frac{1}{A_{m}}$$

where d is the distance between two adjacent laser spots on the camera and $A_{m}$ is the mirror constant. By assuming the volumetric strain of the film is proportional to *z*, the compositional strain ε^⁎^ can be given by [66]:

(4) $$\varepsilon^{*}={\left(1+2.7z\right)}^{\frac{1}{3}}-1$$

During the delithiation–relithiation perturbation at each SOC, the stress change, Δσ, is obtained from the substrate-curvature change, and Δε^⁎^ is obtained from Equation (4), from which the evolution of the biaxial modulus with z can be inferred.

### 2.2. Stress and the Biaxial Modulus in the Lithium-Ion Battery

#### 2.2.1. Potential Stress of Battery Electrode

**Figure 2:**
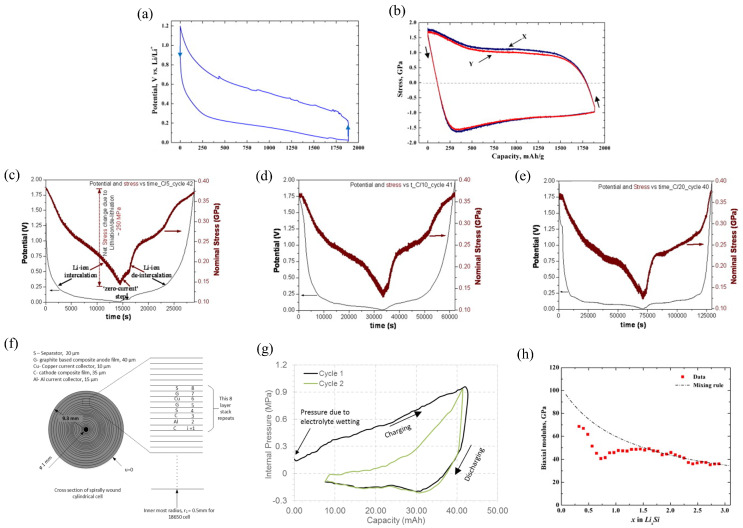
Potential (**a**) and stress evolution (**b**) of the Si thin-film electrode [67]. (**c**–**e**) Combined potential and nominal stress plots of chemical vapor deposition (CVD) carbon films at different charge rates [67]. (**f**) Cross section of the battery and (**g**) the variation in internal pressure [69]. (**h**) Biaxial moduli of silicon thin film [66].

Based on the MOSS measurement system, the curvature changes in the substrate can be obtained by observing the changes in spot spacing of the wafer. Simultaneously, the model enables derivation of stresses generated at the working electrode [67,68]. Figure 2a,b shows the cell potential and the stress evolution of the silicon thin-film electrode in the electro-chemical cycles. In the condition of C/4 rate, the lithium concentration and the stress are assumed to be uniform in the thickness direction. Obviously, lithiation can induce the in-plane expansion of the film, and it is prevented by the substrate. The constraint of the substrate can cause the electrode to undergo elastic deformation initially, followed by plastic strain. This results in compressive stress in the electrode. In the delithiation process, the stress evolution is contrary.

The stress induced in the working electrode during electro-chemical cycling has an obvious impact on the performance of the battery. Reducing the generation of stress can substantially promote the long cycle life of the battery [70]. The variations in capacity and nominal stress measured by the MOSS measurement system at the different rates shown in Figure 2c–e. During film growth and thermal expansion, the CVD carbon film undergoes initial tensile residual stress (0.9 GPa). Due to the irreversibility of the stresses during the initial cycles, the data reported above are from the following cycles, in which reversible stresses can be observed. Obviously, during the galvanostatic cycling, lithiation will induce compression and delithiation will induce tension. It can be concluded that the electrodes at three different rates can have the same maximum compressive stress in the same SOC. Therefore, the CVD carbon electrode can sustain high-rate cycling with minimal degradation. Meanwhile, the compressive stresses observed in the carbon electrode are significantly lower than that of the amorphous Si thin films, which means that the damage to the electrodes during electro-chemical cycling can be reduced. The long cycle life of the battery is substantially increased.

#### 2.2.2. Internal Pressure for Spirally Wound Battery

Based on the electrode induced stresses obtained from the MOSS measurement system, the pressure exerted by electrodes on the battery casing was investigated [69]. The radial displacement, *u*, and radial stress, σ_r_, can be expressed as,

(5) $$u=\frac{1+\upsilon }{1-\upsilon }\frac{\varepsilon^{*}}{r}\int_{r_{i}}^{r}rdr+C_{1}r+\frac{C_{2}}{r}$$

(6) $$\sigma_{r}=-\frac{E}{1-\upsilon }\frac{\varepsilon^{*}}{r^{2}}\int_{r_{i}}^{r}rdr+\frac{E}{1+\upsilon }(\frac{C_{1}}{1-2\upsilon }-\frac{C_{2}}{r^{2}})$$

where *E* and υ are material parameters. *C*_1_ and *C*_2_ are the constant values, *r_i_* is the structure parameter, and ε* is the eigen strain during the lithiation.

The lithium concentration is assumed to be uniform, and the eigen strain in the electrode can be obtained as,

(7) $$\varepsilon^{*}=\sigma (1-\upsilon )/E$$

Equations (5) and (6) can be extended to each layer of Figure 2f, and

(8) $$\sigma_{r}^{L}=\sigma_{r}^{L+1}$$

(9) $$u^{L}=u^{L+1}$$

The cross section of the battery is represented in Figure 2f, and the spirals are approximated by concentric circles. This is reasonable because the thickness of any individual layer is relatively small. The internal pressure of the battery casing varies with capacity, as shown in Figure 2g. It varies continuously with capacity and increases to a peak value (ca. 1 MPa) at the end of charging. The discharge process reverses this. The pressure evolution during the second charge process is lower than the first cycle, from which it can be assumed that the peak pressure in the following cycles will be smaller.

#### 2.2.3. The Biaxial Module of the Silicon Thin-Film Electrode

The result of biaxial moduli of the Si electrode was investigated by using the MOSS, which involved the SOC changes in the anode through electro-chemical cycling, as shown in Figure 2h [66]. The experiments show that the biaxial modulus decreases significantly with an increase in the capacity and the DFT calculation is greatly consistent with the experimental measurement results, which shows that the elastic constants of amorphous Li–Si are well expressed by a simple mixture rule.

This suggests that the method could also be applied to estimate other important mechanical properties, such as ductility and fracture toughness, which are crucial for the realistic modeling and predicting the cycle life of the battery. This means that the same approach could be extended to study and understand the mechanical properties of different electrode materials used in lithium-ion batteries, broadening its potential applications in battery modeling development.

### 2.3. Applications of the Moss Measurement System in Battery Materials

#### 2.3.1. Phase Transformation in Si Wafer

**Figure 3:**
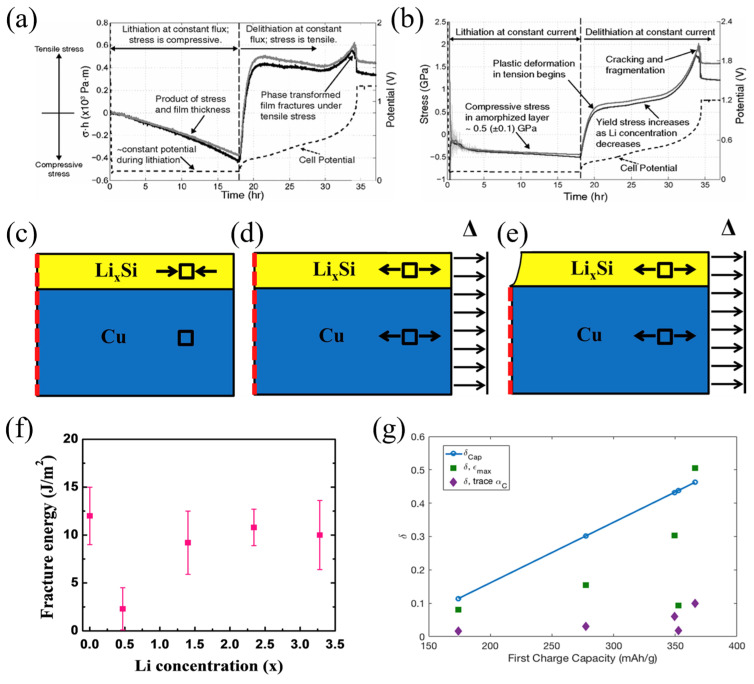
Evolution of cell potential (dashed line), “stress thickness *σh*” (**a**), and pure stress (**b**) with time in the amorphized layer [72]. An illustration (cross-section view) of the ABAQUS simulation to calculate the fracture energy. (**c**) A predefined field (equal biaxial compression) is prescribed in the film to represent the stress due to sputtering, lithiation, and “relaxation”. (**d**) A displacement is applied to the right edge of the film and substrate to represent the bending-induced strain prior to fracture. (**e**) A channel crack is introduced into the film. A predefined field is prescribed in the film to represent the stress [73]. (**f**) Fracture energy as a function of lithium concentration [73]. (**g**) The estimations of oxygen vacancies.

Si is a potential material for increasing the performance of lithium batteries. Understanding the stress evolution and mechanical damage of electro-chemical cycles is critical. In previous works, the adverse effect on the electrodes due to the phase transformation of the Si wafers was investigated. However, detailed information is still lacking. Based on the MOSS, Chon et al. proposed the quantitative relationship between stresses and silicon phase transformation [71]. Crystalline Si (c-Si) and amorphous Li_x_Si (a-Li_x_Si) have a distinct phase boundary. A TEM image reveals that the phase transition takes place in a very narrow region. As depicted in Figure 3a,b, the potential reaches a plateau during lithiation, which indicates that the phase boundary is moving. Since the film thickness here is given by (1 + 2.7z), which increases linearly with charge process, the near-linear changes in *σh* imply that the biaxial stress in the amorphous layer is approximately constant. During lithiation, the stress in the amorphized layer is compressive and rapidly becomes tensile in the delithiation process. The film shows a sudden drop in stress; this is because of the plastic deformation and the fragmentation of the amorphous layer. Moreover, the measurement reported here provides valuable data to build mechanical models to predict damage in the silicon-based anodes.

#### 2.3.2. Fracture Energy of the Si Electrode

Lithium batteries undergo volume expansion and contraction during electro-chemical cycling. The most important strategy to improve battery life and performance is to avoid the fracture of the electrode. The MOSS measurement system can supply the stress and moduli for the measurement of the fracture energy by using the bending test [73]. The strain in the substrate can be obtained by performing a bending test of the working electrode after electro-chemical cycling. Meanwhile, the facture condition of the electrode surfaces can be investigated by transferring to a focused ion beam chamber.

However, the fracture toughness of the electrode cannot be obtained by the critical strain of the substrate. The conventional methods of calculating fracture energy are not suitable for the plastic substrate. Here, an improved model was proposed by performing finite element analyses [73]. The geometry of the simulation is shown in Figure 3c–e, where the steady-state energy release rate is related to the change in internal energy upon introducing the crack. The energy available for fracture of the film can be given as:

(10) $$G=\frac{2(U_{no-crack}-U_{crack})}{h_{f}}$$

where *h_f_* is the thickness of the film and U is the energy of the system. The factor of two in Equation (10) is introduced; this is because only half of the model was simulated. Following standard practice, the pre-factor Z is then calculated using

(11) $$G=\frac{Z\sigma_{f}^{2}h_{f}}{\bar{E_{f}}}$$

where σ_f_ is the critical total stress in the film to cause cracking and ${\bar{E}}_{f}=E_{f}/(1-v_{f}^{2})$ is the plane-strain modulus of the film, where v_f_ is Poisson’s ratio of the film.

Combining Equations (10) and (11) yields:

(12) $$Z=\frac{2{\bar{E}}_{f}(U_{no-crack}-U_{crack})}{\sigma_{f}^{2}h_{f}^{2}}$$

Assuming the substrate to be elastic, this method was verified in ABAQUS [73]. The fracture energy of the pure silicon film is Ѓ = 12.0 ± 3.0 Jm^−2^, as shown in Figure 3f. The lithiated and pure silicon have almost the similar fracture energy, which is consistent with the results of the brittle fracture morphologies in the bending experiments. The results can help design high-capacity batteries without crack formation.

#### 2.3.3. Oxygen Vacancy Measurement by MOSS

Layered lithium transition metal oxides are promising cathode materials for lithium batteries, due to their high energy density [74]. However, during electro-chemical cycling, these materials will suffer from structural changes that lead to substantial voltage fade, and the irreversible stress during delithiation is likely due to oxygen loss. Leah et al. proposed a method to track the irreversible changes in the ${Li}_{1.2}{Mn}_{0.55}{Ni}_{0.125}{Co}_{0.125}O_{2}$ (LR-NMC) cathodes, based on the stress obtained by the MOSS measurement system. The stress measurement and DFT-predicted chemical strain of oxygen vacancies were compared to confirm that the loss of oxygen can induce compression in the electrode. It is assumed that during the activation process, oxygen and lithium are removed in a 1:2 ratio, such that

(13) $$\varepsilon^{*}=\sigma (1-\upsilon )/E$$

(14) $$\delta_{cap}=\frac{x_{Li}}{2}$$

where $\delta_{cap}$ is oxygen capacity and $x_{Li}$ is lithium vacancy concentration.

The non-stoichiometry upper limit value of the oxygen can be given by this method, while the lithium removed along the plateau is accompanied by oxygen loss.

Using the strain per *δ*, which can be obtained by the chemical expansion coefficient tensor, and the strain, the oxygen vacancy concentration can be given as:

(15) $$\delta_{strain}=\varepsilon /\alpha_{C}$$

By using the chemical expansion coefficient tensor, the average strain-predicted vacancy concentration can be obtained, and the upper limit of the strain-prediction values of the oxygen vacancy concentration is also calculated.

The estimations of oxygen vacancies can be obtained based on the measured stress, as illustrated in Figure 3g. Obviously, most of the strain-derived values are smaller than the estimated values, suggesting that the method of assuming all Li removal is accompanied by oxygen loss provides an upper bound on oxygen loss.

This result can be explained by several factors. Firstly, the actual oxygen loss is less than the assumed; secondly, the additional loss of lithium is likely causing a decrease in volume; thirdly, the relaxation of the edges along the crack faces in these films tends to reduce the stresses. Other possible factors include the participation of oxygen in the redox reactions [75], the impact of non-dilute vacancies, or a lower elastic modulus in the delithiated state. Meanwhile, the measurements of oxygen loss on particles are less sensitive compared to the thin films [76]. Table 2 illustrates the value of stress, modulus, and strain in different materials through the MOSS.

## 3. Digital Image Correlation (DIC)

### 3.1. Experimental Device and Mathematical Model

**Figure 4:**
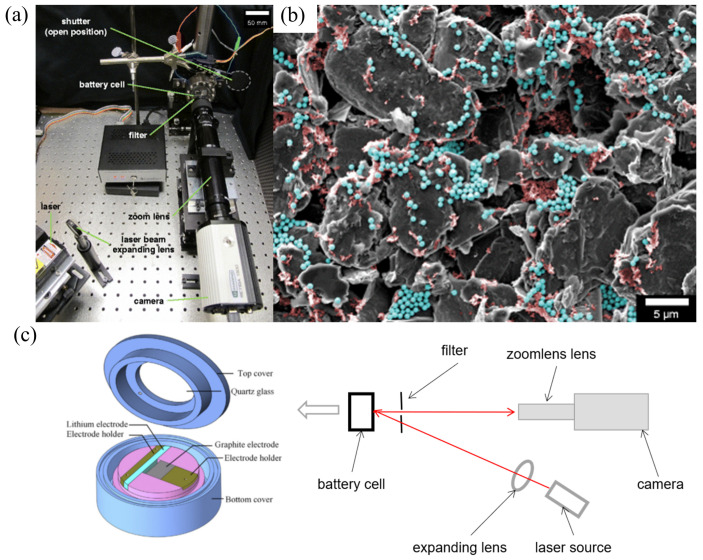
(**a**) Complete imaging device with battery, which consists of a 532 nm laser, reflected laser light, shutter blocks, and so on [29]. (**b**) SEM micrograph of a composite graphite electrode, where carbon black can be seen in red while the silica nano-particles are in blue [29]. (**c**) Schematic diagram of the DIC device for measuring strain. Here, graphite is the negative electrode material, and Li metal is the cathode electrode material.

The DIC system employs a camera equipped with a zoom lens to record fluorescence speckle patterns from the electrode’s surface, which are illuminated by laser light throughout the battery’s electro-chemical cycling [9,28]. High magnification reveals natural spot patterns in composite graphite electrodes ideal for DIC analysis. However, variations in lithium content during cycling alter the color of graphite, causing transformations in the appearance, size, and shape of these spots due to particle deformation and movement. These changes have led to poorly correlated speckle patterns, as shown in Figure 4a [29].

To enhance visibility, fluorescent particles are added to the electrode surface, which, when observed under SEM, display carbon black and fluorescent silica in red and blue, respectively (Figure 4b). Fluorescence spot patterns, thus, allow for the deduction of lithium-ion content variations, facilitating the calculation of displacement and deformation [29]. By using code to process the image, the actual displacement can be calculated through code and formulas, and strains are calculated by interpolating displacements using finite element shape functions.

### 3.2. Strain of the Graphite Electrodes

**Figure 5:**
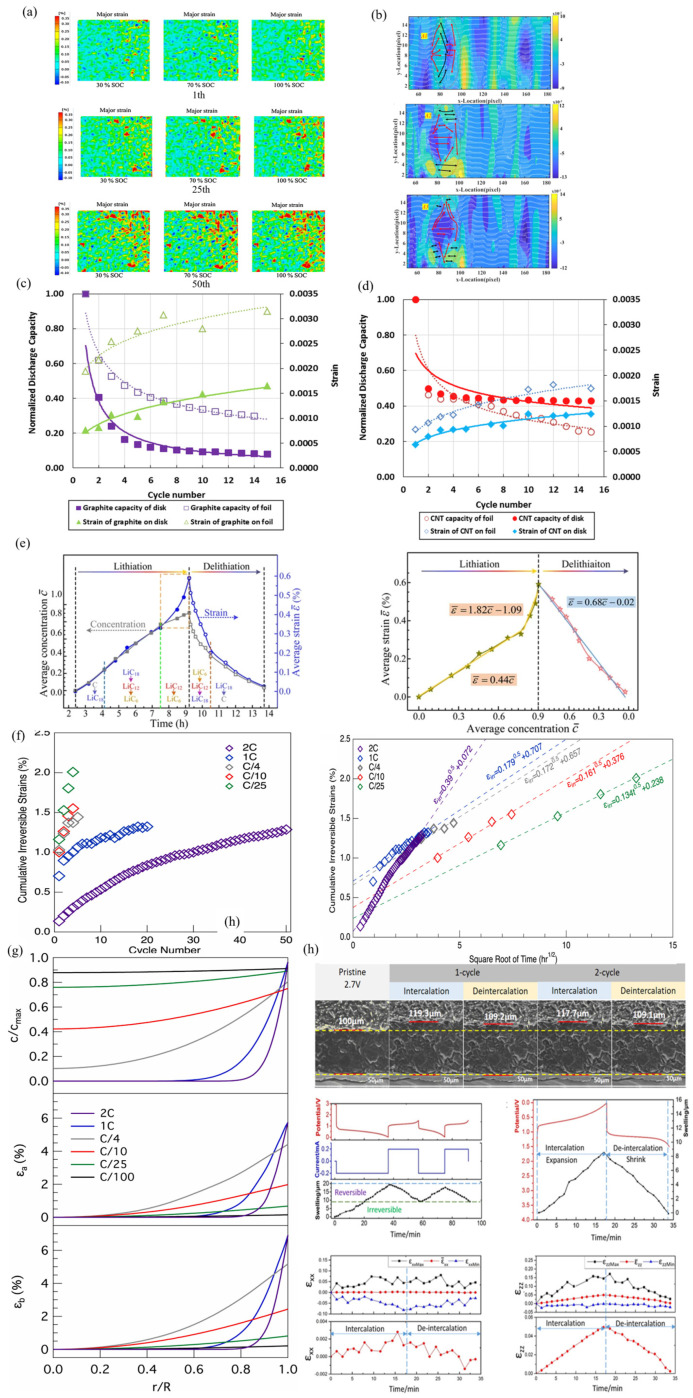
(**a**) The evolution of principal strain in the lithium-ion cell at different SOCs with different cycle numbers (1th, 25th, 50th) [81]. (**b**) Lithiation process with variable current densities. The in-plane maximum Principal strain distributions at the SOC of (A1) 5% with the current density of 0.025 mA cm^−2^, (A2) 31% with the current density of 0.075 mA cm^−2^ and (A3) 67% with the current density of 0.125 mA cm^−2^ [78]. (**c**,**d**) Normalized discharge capacity and corresponding strain for a graphite electrode and CNT-based electrode [29]. (**e**,**f**) The connection between the strain and concentration of the graphite electrode at different times and cycles [77]. (**g**) Cumulative irreversible strains in the composite NFP electrode cycled at different rates [79]. (**h**) Na concentration and misfit strains in electrode particle at five different scan rates [80].

The evolution of principal strain in the x–y plane at 30%, 70%, and 100% SOC at different numbers of cycles was investigated [77]. The principal strain seemed to change little in the 1st cycle. However, as the number of cycles increased, the change in principal strain rapidly increased. By the 25th charging cycle, it was found that the strain on the battery’s top right side exceeded that on the opposite side. This phenomenon is obvious in Figure 5a, where the maximum major strain on the right side of the battery is greater than 0.35%, and the major strain on the upper-right side of the battery is the maximum. Li et al. clearly illustrated the process of ions penetrating the entire SE [78]. Under the action of external voltage, the stress caused by diffusion compresses the SE lattice through lithium-ion migration, causing deformation of the enclosed areas A1, A2 and A3 (Figure 5b). This result indicates that the permeation of lithium ions can be hindered, resulting in a charge in the penetration direction. The current density and inhomogeneity determine the penetration direction. Regions with strong local current density result in greater diffusion-induced stress. In addition, mechanical inhomogeneity changes the penetration path to a certain extent, forcing ions to bypass and seek paths with lower mechanical resistance. As cycles increase, residual strain within the working electrode rises, leading to a decrease in battery capacity (Figure 5c,d). For the graphite electrode specimens, the larger the strain difference, the greater the corresponding discharge capacity difference. For the narrower strain difference found in CNT-based electrodes, the discharge capacity curve is closer. The strain difference of the CNT shell is small, and the total strain amplitude is low, which can be attributed to the high stiffness of the CNT composite film Among these two types of electrodes, the discharge capacity loss rate of the foil substrate sample is higher after more than 8 cycles [29]. Figure 5e shows the average concentration, $\bar{c}$, and average strain, $\bar{\varepsilon }$, at different times during the lithium insertion and lithium removal processes. The experimental results indicate that, during the lithiation process, the concentration and plane strain both increase nonlinearly, with the former increasing rapidly and then slowly, while the latter initially increases slowly and then rapidly. The correlation between concentration and in-plane strain evolution indicates that a high strain always corresponds to a high concentration, although the two were not always linearly correlated and accompanied by segmentation features. Therefore, experimental analysis inferred that high in-plane strain suppressed diffusion and lithiation. The experimental data clearly indicated that the evolution of in-plane strain with concentration was not completely linear (Figure 5e).

To distinguish these factors at different rates of irreversible strain, a graph was drawn showing the relationship between cumulative irreversible strain and the number of cycles, as shown in Figure 5f. If the dominant force on the irreversible deformation is the dissolution of iron metal from the NaFePO_4_ structure, negative irreversible strain is expected to be observed with increasing cycles. Another factor that leads to irreversible deformation may be the formation of vacancies in the electrode crystal structure. Figure 5g proved that large deformation occurs only in the first cycle of the sodium iron phosphate cathode at all scanning rates. In addition, a mathematical model based on continuous media was used to predict the growth rate of solid electrolyte interface layers with time square roots [79]. The strain rates related to capacity are related to the generation of mismatch strains in the electrode. Here, the transfer model only simulates a rate-induced concentration gradient in solid solution, as shown in Figure 5g. This model predicts that the concentration gradient of sodium near the electrode surface will be higher, which will lead to a greater mismatch strain. It should be noted that a large concentration gradient can hinder the volume mismatch between two independent phases in the electrode [79].

The process of cross-sectional changes was monitored using in situ SEM technology, and the initial thickness of the graphite-based electrode was 100 μm, as shown in Figure 5h. During the lithiation of the first cycle, electrode expansion and increased thickness were discovered. In the second cycle, the thickness of the expanded electrode was between 117.7 μm and 109.1 μm. This means that the expansion deformation of the electrode is completely restored. Through DIC analysis, the changes in average electrode thickness and voltage over time during the two cycles were obtained on the strain values of the x and z directions during the second cycle; the evolution curves of the maximum strain, minimum strain, and averaged strain over time in the calculation region are shown in the Figure 5h. Expansion strain occurred simultaneously in both directions. According to the quantitative description of the average strain, the macroscopic deformation of the electrode exhibited anisotropic characteristics [80].

### 3.3. Lithium-Ion Concentration and Stress

**Figure 6:**
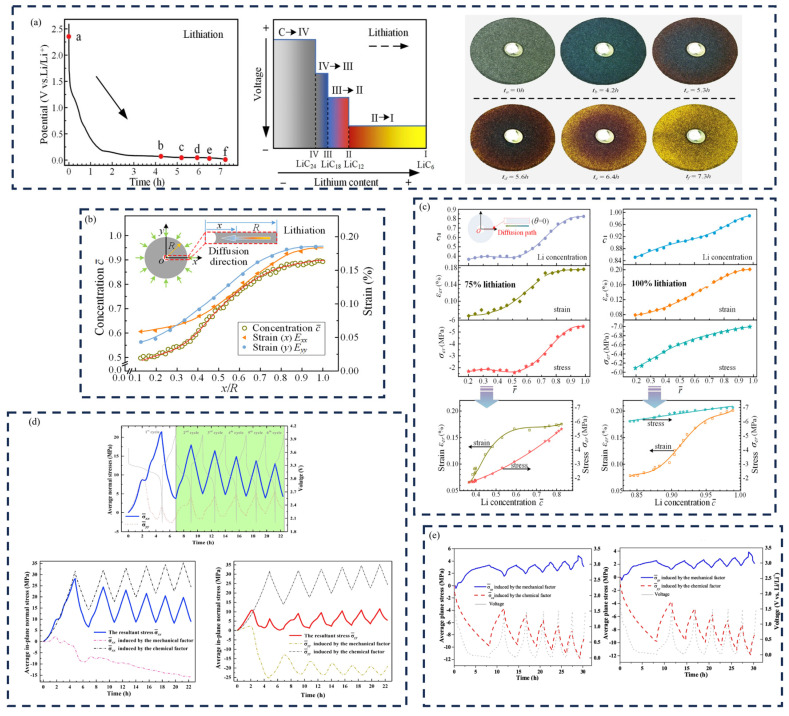
(**a**) Potential and color evolution of a graphite electrode during the first cycle. There is an obvious potential platform below 0.2 V, which corresponds to the formation of the phase and the change of color [82]. (**b**) Distribution of concentration and strain along diffusion direction at t = 6.4 h [82]. (**c**) Distributions of Li+ concentration, strain, and stress of the graphite electrode at 75% and 100% lithiation. (**d**) Dynamic change in average in-plane normal stresses, σ_xx_ and σ_yy_, as a function of test time [83]. (**e**) Chemical and mechanical effects on the stress variation in SiO@C composite electrodes [84].

Figure 6a illustrates the potential evolution and associated color changes in the graphite electrode during its first cycle, revealing a clear correlation with the strain field [82]. To explore the intricate interplay between concentration and strain, Figure 6b shows E_xx_ and E_yy_, the diffusion path, along the intercalation time of 6.4 h (y = 0). These results underscore a consistent alignment in the distributions of strain and concentration. Radially progressing outward from the electrode’s center, a gradual initial increase followed by a rapid ascent and eventual stabilization of values were observed. These findings provide fundamental experimental insights for a nuanced exploration of the impact of deformation on electro-chemical processes within the electrode, laying the groundwork for subsequent in-depth analyses.

Figure 6c presents the lithium concentration, strain, and electro-chemical stress within the graphite electrode. The Li^+^ concentration positively correlates with tensile strain due to the electrode material’s expansion and compression caused by strain mismatch [85,86]. Those responses all exhibit an incremental trend as the electro-chemical process unfolds [87]. Notably, the rate of increase is more pronounced in the interior compared to the exterior. It can be concluded that the mechanical response can affect the diffusion (Figure 6d) [87,88].

Mao et al. also address the average normal stresses, σ_xx_ and σ_yy_, as illustrated in Figure 6e [83]. The variations in σ_xx_ and σ_yy_ are complex, and are attributed to the series of phase transitions of LiXV_2_O_5_ and side reactions [89]. Chemically induced stress increases during the discharge phase and decreases upon charging [90,91]. This phenomenon is attributed to the build-up of residual lithium ions throughout cycling, signifying the amplified peak stresses [30,92]. A comprehensive understanding of the chemical-mechanical coupling mechanism inherent in the SiO@C composite electrode was sought [84]. The average in-plane stress provoked by both mechanical and chemical factors is meticulously calculated and analyzed. Table 3 illustrates the value of stress, strain, and modulus in different composite electrodes through DIC.

## 4. Bending Curvature Measurement System (BCMS)

### 4.1. Experimental Device and Mathematical Model

**Figure 7:**
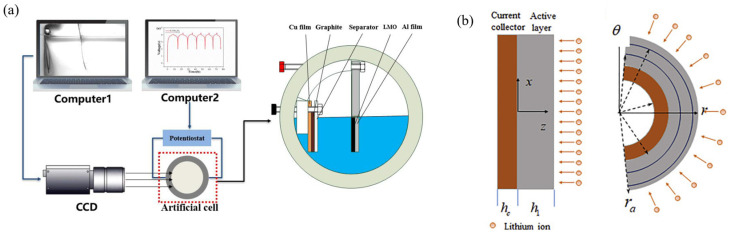
(**a**) Schema of the Bending Deformation Measurement System, which is composed of artificial cells, an image acquisition system and a data analysis system. Computer1 is responsible for collecting the experimental electrode bending deformation pictures taking by the CCD camera in real time. Computer2 is mainly used for electrochemical testing of artificial pictures [95]. (**b**) Structure of the electrode with an active layer and current collector [96].

Quantitative measurement in situ of the electrode properties and induced stress in the composite electrodes is difficult. The bending curvature measurement system (BCMS) method is an effective way to analyze the bending deformation, electrode properties, and stress evolution of the batteries during electro-chemical cycles. A layered electrode structure, as shown in Figure 7a, was devoted to investigate the connection between curvature changes and the cell capacity in the electro-chemical experiments. The observation window of the experimental setup is transparent quartz glass for easy data collection using a camera. The working electrode consists of a composite graphite layer and a copper foil layer, and the reference electrode consists of a lithium metal, while the two electrodes are separated by a separator. The cell was full of 1 M LiPF_6_ salt and the experiment was performed in a high-argon atmosphere. Electro-chemical cycling of the composite graphite electrode was carried out at low current density and room temperature. Based on the BCMS, the deformation data and the bending curvature of the electrode can be extracted [93]. Mechanical analysis provides the chance to analyze the mechanism of electrode deformation. A cantilever beam structure consisting of composite electrodes bonded to copper foils is shown in Figure 7b. Here, $h_{1}$ and $h_{c}$ are the structure parameters. The composite electrode swells and shrinks during the lithiation and delithiation process and the bending curvature of the cantilever beam will become larger or smaller with the restriction of current collector. Here, the *z*-axis coincides with the thickness and the x–y plane coincides with the plate [94].

Under the small deformation theory, the in-plane normal strain can be written as [94,97]:

(16) $$\varepsilon =\varepsilon_{0}+\kappa z$$

where $\varepsilon_{0}$ is the in-plane strain at the plane of z=0 and κ is curvature. Usually, the composite electrode is made up of active materials, a binder, and a conductive agent. For simplification, the active layer is assumed to be macroscopically homogeneous and isotropic. At a comparatively small rate of charge and discharge, we can presume that the concentration c remains constant along the *z*-axis as the electrode undergoes electro-chemical cycling. The equation can be expressed as:

(17) $$\begin{array}{l} \sigma_{1}=E_{1}(c)\left(\varepsilon_{0}+z\kappa \right)-\frac{1}{3}E_{1}(c)\Omega c\\ \sigma_{c}=E_{c}\left(\varepsilon_{0}+z\kappa \right) \end{array}$$

where $E_{1}(c)$ and Ω are the active layer parameters. Presuming the absence of mechanical constraints on the bilayer and the fulfillment of equilibrium in total force, torque balance requires:

(18) $$\int_{-h_{c}}^{h_{1}}\sigma_{x}dz=0\;\int_{-h_{c}}^{h_{1}}\sigma_{x}zdz=0$$

Substituting Equation (25) into Equation (26) yields the strain, $\varepsilon_{0}$, and curvature, κ, of the electrode as:

(19) $$\kappa =\frac{\Omega }{3h_{1}^{3}}\frac{\alpha_{1}}{\alpha }\int_{0}^{h_{1}}czdz+\frac{\Omega }{3h_{1}^{2}}\frac{\alpha_{2}}{\alpha }\int_{0}^{h_{1}}cdz=\frac{2\alpha_{5}\Omega c}{h_{1}\alpha }$$

(20) $$\varepsilon_{0}=\frac{\Omega }{3h_{1}^{2}}\frac{\alpha_{2}}{\alpha }\int_{0}^{h_{1}}czdz+\frac{\Omega }{3h_{1}}\frac{\alpha_{3}}{\alpha }\int_{0}^{h_{1}}cdz=\frac{\alpha_{4}\Omega c}{3\alpha }$$

Here, α–α_5_ relies on the sample structure and mechanical properties (e.g., electrode thickness, $h_{1}$, and copper foil, $h_{c}$, and elastic moduli of the electrode, $E_{1}$ and $E_{c}$). After rewriting Equation (19), a quadratic equation is obtained, including the curvature, modulus ratio $R_{E}=E_{c}/E_{1}$, thickness ratio $R_{h}=h_{c}/h_{1}$, and lithium concentration, c. This equation and its solution are given in Equations (21) and (22):

(21) $$\kappa h_{1}R_{h}^{4}R_{E}^{2}+(4\kappa h_{1}R_{h}+6\kappa h_{1}R_{h}^{2}+4\kappa h_{1}R_{h}^{3}-2\Omega cR_{h}-2\Omega cR_{h}^{2})R_{E}+\kappa h_{1}=0$$

(22) $$R_{E}=\frac{\begin{array}{l} (-(4\kappa h_{1}R_{h}+6\kappa h_{1}R_{h}^{2}+4\kappa h_{1}R_{h}^{3}-2\Omega cR_{h}-2\Omega cR_{h}^{2})+\\ \sqrt{{\left(4\kappa h_{1}R_{h}+6\kappa h_{1}R_{h}^{2}+4\kappa h_{1}R_{h}^{3}-2\Omega cR_{h}-2\Omega cR_{h}^{2}\right)}^{2}-4\kappa^{2}{h_{1}}^{2}R_{h}^{4}}) \end{array}}{2\kappa h_{1}R_{h}^{4}}$$

To obtain the partial molar volume and modulus, this method can be improved, and the connection among curvature with material parameters can be expressed as:

(23) $$R_{h}^{4}R_{Es}^{2}-2R_{h}(3\left(R_{h}+1\right){\bar{\kappa }}^{-1}\varepsilon^{*}-(2R_{h}^{2}+3R_{h}+2))R_{Es}+1=0$$

Owing to electrodes with different thicknesses, the ratio of modulus can be given as:

(24) $$R_{Es}=\frac{p\prime q\prime \prime -p\prime \prime q\prime +\sqrt{{\left(p\prime \prime q\prime -p\prime q\prime \prime \right)}^{2}-(q\prime R_{h}^{\prime \prime 4}-q\prime \prime R_{h}^{\prime 4})\left(q\prime -q\prime \prime \right)}}{q\prime R_{h}^{\prime \prime 4}-q\prime \prime R_{h}^{\prime 4}}$$

where:

(25) $$\begin{array}{l} R_{h}^{\prime }=\frac{h_{1}}{h_{c}},R_{h}^{\prime \prime }=\frac{h_{2}}{h_{c}},p\prime =R_{h}^{\prime }(2+3R_{h}^{\prime }+2R_{h}^{\prime 2}),q\prime =R_{h}^{\prime }\left(1+R_{h}^{\prime }\right){\bar{\kappa }}^{-1}\\ p\prime \prime =R_{h}^{\prime \prime }(2+3R_{h}^{\prime \prime }+2R_{h}^{\prime \prime 2}),q\prime \prime =R_{h}^{\prime \prime }\left(1+R_{h}^{\prime \prime }\right){\bar{\kappa }}^{-1} \end{array}$$

A camera was employed to capture the curvature variations in the electrodes of varying thicknesses, while a micrometer quantified the thickness parameter (h1, h2, and hc). Utilizing these curvature and thickness values, Equation (24) can be solved to ascertain the evolution of elastic modulus with respect to SOC. This equation underscores the interdependence between the electrode curvature and material performance parameters. The expression for the eigen strain of a composite electrode during electro-chemical cycling is articulated as follows:

(26) $$\varepsilon^{*}=\frac{R_{h}^{\prime \prime 4}p\prime -R_{h}^{\prime 4}p\prime \prime }{3\left(R_{h}^{\prime \prime 4}q\prime -R_{h}^{\prime 4}q\prime \prime \right)}+\frac{\left(R_{h}^{\prime 4}-R_{h}^{\prime \prime 4}\right)\left(p\prime q\prime \prime -p\prime \prime q\prime -\sqrt{{\left(p\prime \prime q\prime -p\prime q\prime \prime \right)}^{2}-(q\prime R_{h}^{\prime \prime 4}-q\prime \prime R_{h}^{\prime 4})\left(q\prime -q\prime \prime \right)}\right)}{6\left(q\prime -q\prime \prime \right)\left(R_{h}^{\prime \prime 4}q\prime -R_{h}^{\prime 4}q\prime \prime \right)}$$

By combining Equations (24)–(26), the partial molar volume of the composite electrode can be calculated with

(27) $$\Omega_{composits}=\frac{{\left(1+\varepsilon^{*}\right)}^{3}-1}{c}$$

Stress measurements during electro-chemical cycling of the composite electrode can also be measured by combining Equations (17), (19) and (20). Different from the MOSS system, the bending curvature measurement system can be used to monitor the connection between the curvature (k) and the state of charge (SOC) of a thicker active layer in the electro-chemical experiments and the mechanical properties and stress evolution in composite electrodes can be analyzed effectively [97].

### 4.2. Modulus and Stress

**Figure 8:**
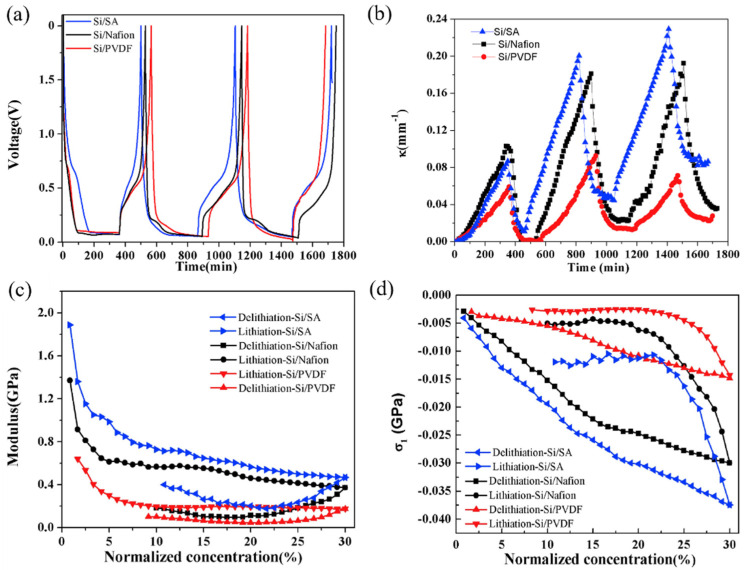
Voltage (**a**) and curvature evolution (**b**) for the first 3 cycles of the silicon composite electrodes, and the modulus (**c**) and stress evolution (**d**) under the second cycle [98].

Based on the BCMS, a comprehensive analysis of the composite electrodes was conducted to obtain the parameters for the electrode, such as the curvature, modulus, and induced stress. The voltage and curvature evolution of the silicon composite electrodes composed of different binders (i.e., SA, Nafion, and PVDF) are shown in Figure 8a,b. Evidently, the curvature in all three electrodes follows a pattern of increase during lithiation and decrease during delithiation. Notably, the curvature evolution of the composite electrode with a SA binder surpasses that of Nafion, which is the second largest, and PVDF, the smallest, across the entire capacity range. This observation also highlights that the initial cycle exhibits relatively minor curvature variation compared to subsequent cycles. This phenomenon is attributed to the formation of a solid electrolyte interface (SEI), which consumes a substantial amount of lithium [98]. In the third cycle, the curvature changes in both the Si/SA and Si/Nafion composites are comparable to those in the second cycle, owing to identical lithiation capacities [99]. However, in the case of the silicon composite electrode with a PVDF binder, the curvature alterations in the third cycle are smaller than those in the second cycle, a result attributed to the formation of cracks.

Here, the curvature data of the more stable second cycle were extracted for modulus and stress calculations; the evolution of modulus and induced stress with lithium concentration of the silicon composite electrode are shown in Figure 8c,d. It is imperative to understand that the modulus alteration described herein reflects an average value for the complete composite electrode, incorporating the coupled impact of both porosity and volume expansion. Significantly, all composite electrodes, regardless of the binder used, exhibit a decreasing trend during lithiation, indicative of the softening of silicon particles. Meanwhile, composite electrodes with different binders show different trends of modulus decrease, which can be explained with the different elastic moduli and volume fractions of constituents, porosity, and cracks. The three different composite electrodes here also perform similar trends and different trends in the stress evolution during electro-chemical cycling. Each electrode type experiences an increase in compressive stress as lithium concentration rises during lithiation, with Si/SA and Si/Nafion electrodes showing a notably higher increase compared to Si/PVDF electrodes. The observed increase in the magnitude of compressive stress during lithiation is explained by the fact that a higher lithium concentration, as Si takes in more lithium ions, leads to a larger volumetric strain. On the flip side, the changes in both modulus and compressive stress during the delithiation stage are diminished, a phenomenon believed to stem from crack formation occurring in the delithiation process.

**Figure 9:**
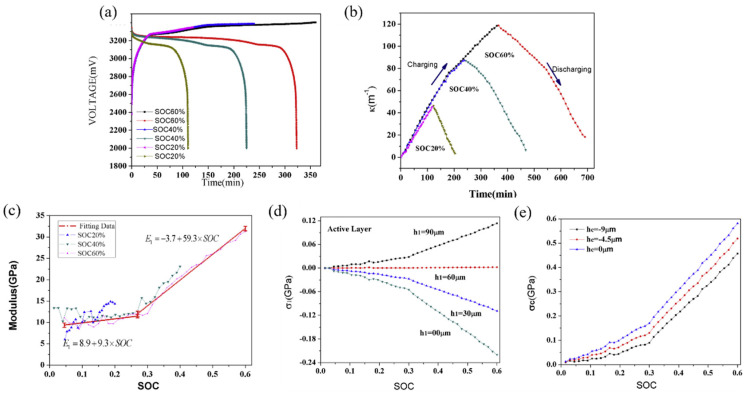
Voltage (**a**), curvature (**b**), elastic modulus (**c**), and stress (**d**,**e**) evolution of the graphite composite electrode [31].

Based on the BCMS, the voltage and curvature evolution of the graphite electrode under different SOCs are shown in Figure 9a,b. Regardless of varying SOC levels, the curvature alterations in the graphite electrode exhibited consistent trends: a nearly linear increase during charging and decrease during discharging. By substituting the curvature changes, thickness parameters, and modulus of the electrode into the mathematical model, the variations in elastic modulus and induced stress of the graphite composite electrode during electro-chemical cycling can be analyzed, as expressed in Figure 9c–e. In contrast to silicon composites, the elastic modulus of graphite electrodes increases gradually at first and then more rapidly as the SOC rises. Initially, the modulus increases from a value of 8.9 GPa to 11.9 GPa between a SOC of 0% and a SOC of 27%, with a trend of *E*1 = 8.9 + 9.3 × SOC, then increases from 11.9 GPa to 31.9 GPa when the SOC is 60% with a trend of *E*1 = −3.7 + 59.3 × SOC. It can be seen that the speed of growth of the elastic modulus is related to the value of the SOC and the delithiation process reverses.

Furthermore, modulus changes significantly influence the overall deformation rate of the electrode. A changed modulus in the graphite electrode correlates with a deceleration in the deformation process, leading to a diminishing slope, as depicted in Figure 9b. Throughout the lithiation phase, stress in both the active layer and the current collector demonstrates an almost linear escalation relative to capacity, progressing in the direction of thickness. In general, compressive stress is produced in the active plate near the interface due to lithiation-induced expansion and restriction by the current collector. While at the outer layer, the stress undergoes a transition from compressive to tensile. A stress-free layer is pinpointed at the position *h*_1_ = 60 μm within the electrode, arising from the interplay between diffusion-induced stress and bending stress, as illustrated in Figure 9d. As more lithium ions migrate into the electrode, the curvature undergoes a pronounced increase, amplifying the significance of bending and resulting in a convex curvature at the outer layer. Consequently, compressive stress transitions into tensile stress. In the current collector, tensile stress is evident, with smaller stress in the outer layer (−9 μm) and larger stress in the inner layer (0 μm), as shown in Figure 9c. In contrast, the composite graphite layer experiences concurrent compressive and tensile stress. Notably, these findings align with the theoretical predictions presented in prior research.

### 4.3. Partial Molar Volume

**Figure 10:**
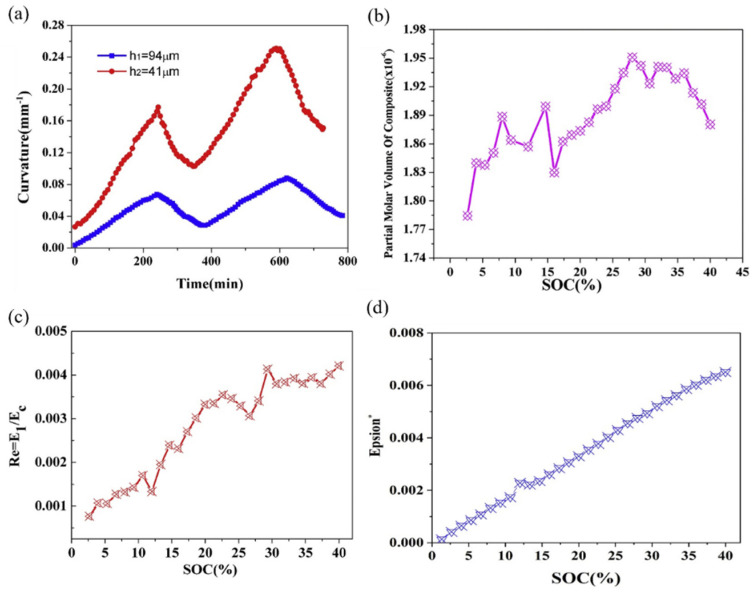
Evolution of curvature (**a**), partial molar volume (**b**), modulus (**c**), and strain (**d**) of a composite graphite electrode corresponding to a SOC in the 2nd cycle [96].

Throughout the lithiation process, lithium ions consistently penetrate the composite electrode, inducing the expansion of the active layer [100]. Curvature evolution of the graphite electrodes with different thicknesses increases greatly during lithiation as shown in Figure 10a. The curvature linearly increases as lithium ions continuously infiltrate the composite electrode, elevating stress within the active layer and causing a progressive bending of the electrode during lithiation; this process reverses during delithiation. Moreover, thicker active layers exhibit reduced bending deformation compared to their thinner counterparts. This phenomenon can be attributed to the constraints imposed by the current collector. Based on the BCMS, the variation in partial molar volume can be determined, as expressed in Figure 10b. The reported partial molar volumes represent average values of the composite electrode, rather than the graphite material alone. Throughout the lithiation process, the partial molar volume of the active layer exhibits an almost linear increase against the capacity before a SOC of 28%. Subsequently, the results plateau and demonstrate a decreasing trend between 28% and 40% SOC. This can be explained by the in situ XRD analysis, which reveals that graphite undergoes a phase transformation, corresponding to the plateau observed between Li_0.25_C_6_ and Li_0.5_C_6_, and the observed tendency in molar volume change aligns with this phase changing.

This breakthrough in in situ experimental techniques and theoretical modeling marks a significant advance in determining composite electrodes’ partial molar volume during cycling. The computation of modulus and eigen strain involved the concentration, curvature changes, and thickness parameters, and the investigation of modulus ratio and swelling strain with lithium concentration in graphite composite electrodes during the second cycle is presented in Figure 10c,d. The elastic modulus ratio of the composite electrode significantly increases during the second lithiation, and the results show that the graphite composite electrode hardened remarkably, while the state of charge increased. Meanwhile, the strain values exhibit a linear increase, reaching 0.007 at a state of charge (SOC) of 40%, corresponding to a volumetric expansion of 1.9%. It should be known that this expansion is less pronounced than that observed in pure graphite (10% when fully lithiated), and can be attributed to the porous microstructure characteristic of commercial graphite electrodes [101].

**Figure 11:**
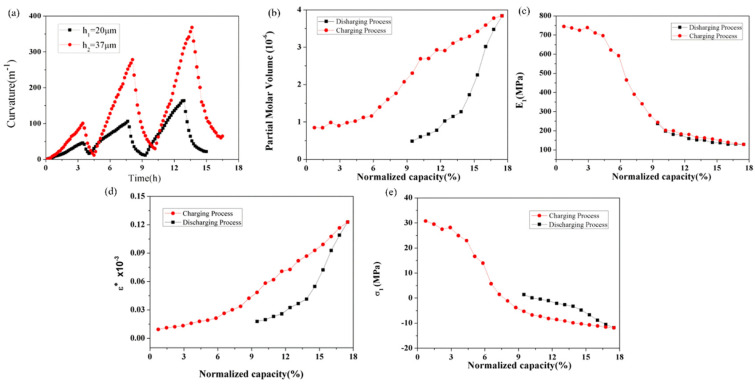
Evolution of curvature (**a**), partial molar volume (**b**), modulus (**c**), strain (**d**), and induced stress (**e**) of the silicon composite electrode corresponding to the SOCs in the 2nd cycle.

Based on the BCMS, the curvature evolution of the silicon electrode can be obtained. The curvature increases linearly during the lithium intercalation process, and reverses during the delithiation process, as shown in Figure 11a. Similar to the graphite composite electrode, in the first three cycles, the maximum curvature shows an increasing trend, during which the formation of SEI irreversibly consumes a large amount of lithium ions. Furthermore, the thickness affects the bending curvature of electrodes during the cycle, as the thicker Si electrode has a greater curvature than the thinner Si electrode during the same charging stage. Further, the partial molar volume and elastic modulus of the silicon electrode were calculated by mathematical model, and are shown in Figure 11b,c. The partial molar volume increased and decreased during the lithium insertion and removal processes, respectively. Here, the partial molar volume of the Si composite electrodes is different from that of Si itself, as the composite electrodes have small pores, conductive agents, and binders. Meanwhile, the elastic modulus was 100 Mpa in the 17% SOC. By combining the electrode parameters and mathematical model, the variations in strain and induce stress with the charging state were derived, as illustrated in Figure 11d,e. During the lithium insertion process, the volume expansion of the active material is constrained by the copper foil, resulting in a linear increase in strain. Subsequently, the strain decreases linearly during the delithiation process. Furthermore, there is an initial presence of tensile stress, approximately 31 MPa, in the Si composite electrode at the beginning of the second lithiation, which progressively diminishes throughout the subsequent lithiation stages [102]. The transition from tensile to compressive stress occurs around a normalized capacity of 7%. During the lithium removal phase, compressive stress decreases, shifting back to tensile stress at 10% normalized capacity and reaching approximately 2 Mpa. These mechanical property data and outcomes are valuable for the advancement of battery models.

**Figure 12:**
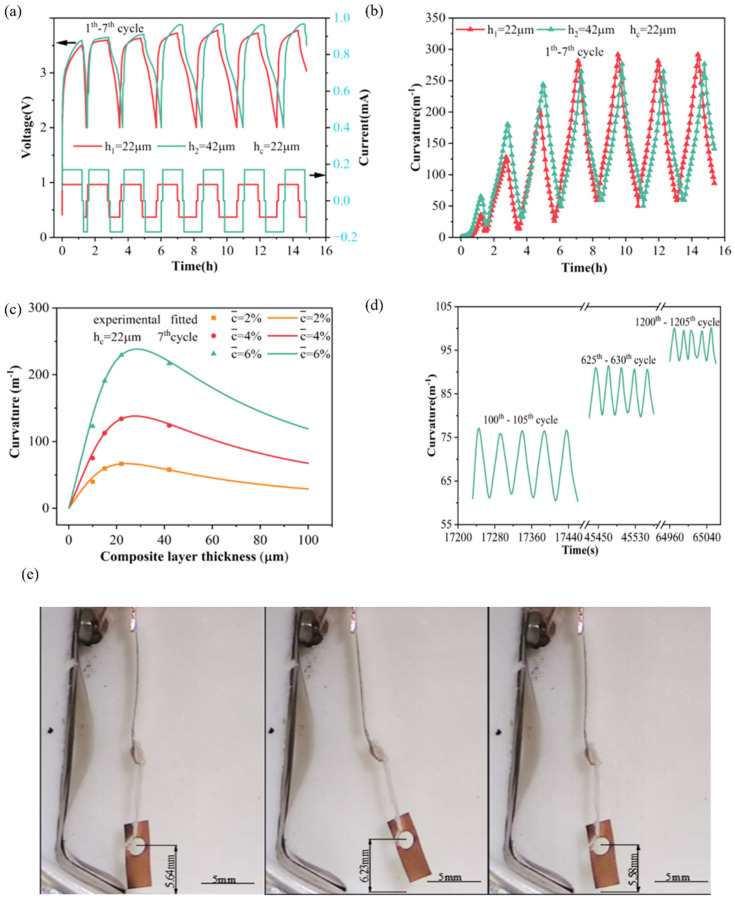
(**a**) Voltage-current and (**b**) measured curvatures of the silicon composite electrode. (**c**) Prediction results of curvature changes under different electrode thicknesses. (**d**) Long-cycle testing and (**e**) loading testing of silicon composite electrodes [93].

Electro-chemical actuators can transform electrical energy into mechanical energy directly. Based on the BCMS, the controllability of electrode deformation can be realized [96]. Figure 12a–c displays the voltage, measured curvatures, and predicted curvatures for silicon composite electrodes with varying thickness ratios. Integrating the BCMS’s mathematical model with deformation curvatures from electrodes of two thicknesses allows the calculation of material parameters, including elastic modulus, partial molar volume, and stress evolution. Therefore, the curvature changes under different electrode thickness ratios can be predicted, as shown in Figure 12c. The realization of the deformation controllability of composite electrodes promotes the application of electro-chemical actuators. Moreover, the silicon composite electrode-based electrochemical actuator exhibits consistent driving performance through extensive testing cycles, with bending curvatures maintaining stable values, as demonstrated in Figure 12d. This excellent phenomenon indicates that the Si/CNTs composite-based actuator has good durability and working performance, giving potential for development. Additionally, load testing was conducted, involving a copper sheet suspended by a separator-made string at the actuator’s end and the electro-chemical actuator successfully lifted a load equal to its weight, as shown in Figure 12e. This experiment demonstrates that the electro-chemical actuator has an effective loading capacity. Table 4 illustrates the values of stress, strain, and modulus in different composite electrodes through the BCMS.

## 5. Conclusions

**Figure 13:**
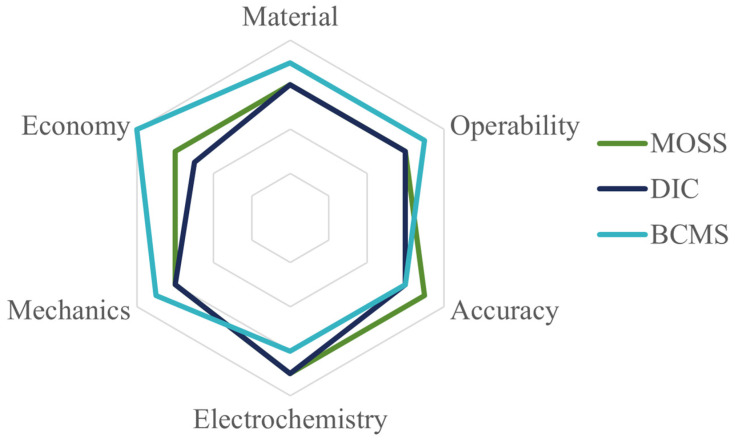
The radar chart of three different in situ methods.

There are multiple problems that can trigger the degradation of lithium batteries. Understanding the electrode mechanism and the performance variation in the batteries during the electro-chemical cycles can allow the effective prediction of the degradation of the batteries. In this review, we introduce three in situ measurement systems, namely the multi-beam optical stress sensor (MOSS) measurement system, the digital image correlation (DIC) measurement system and the bending curvature measurement system (BCMS), in terms of experimental equipment, measurement mechanism, description of the experimental results, and related research. The radar chart of three different in situ methods is shown in Figure 13, which gives readers a direct comparison to help choose which in situ measurement method to use. It can be observed that the BCMS can be applied to any composite material at a low cost, while the MOSS shows the highest accuracy, and DIC is even better in the electro-chemical detection section.

Through an overview of the research on different test systems, we can analyze the unique characteristics, advantages, and disadvantages of the different systems. The MOSS can be used to measure the distribution of electrode stress without contact, which will prevent contact damage to the battery, while having high-precision, accurate, and reliable measurement results. However, the laser source and optical device cannot be disturbed during the testing, which places a high demand on the measurement environment and involves expensive test setups. DIC has a unique advantage in using image-acquisition and image-processing algorithms to monitor the deformation and stress evolution of the electrode, especially in the processing of graphite electrodes, and can provide high-resolution measurement results. However, due to the high-quality image-acquisition and complex-processing algorithm requirements, the sensitivity of the system-measurement process is relatively low, and there may be measurement deviation in the recognition of deformation and strain in local areas. Alternatively, the BCMS can be used to obtain the curvature value by photographing the deformation of the electrode during electro-chemical cycles, and the corresponding composite material parameters can be calculated with the help of the model. Although the measurement accuracy is slightly lower than that of the other two systems, the calculation process is simpler, easier to operate, and applicable in more scenarios than the other two systems, which grants more application prospects. This review summarizes three mainstream systems in battery performance testing, which has great guiding value for improving battery performance and creating the next generation of lithium batteries.

## Figures and Tables

**Table 1:** Comparative summary of in situ characterization tools for battery electrode analysis.

Characterization Tool	Characteristic Length Scale	Pros	Cons
Multi-beam optical stress sensor (MOSS)	Nanometers to micrometers	Non-contact, high precision	Sensitive to disturbances, expensive
Digital image correlation (DIC)	Micrometers to millimeters	High-resolution deformation measurement	Requires high-quality imaging and complex processing
Bending curvature measurement system (BCMS)	Micrometers to centimeters	Simple operation, applicable in diverse scenarios	Slightly lower accuracy, indirect method for mechanical properties
Scanning electron microscope (SEM)	Nanometers to micrometers	High-resolution imaging	Vacuum operation, may alter native state, not for real-time analysis
Atomic force microscopy (AFM)	Nanometers	High spatial resolution, measures nanoscale mechanical properties	Limited sample area, slow, requires careful operation
Transmission electron microscopy (TEM)	Atomic to nanometers	Highest spatial resolution, comprehensive material characterization	Complex sample prep, vacuum operation, limited field of view, not for in situ mechanical measurements

**Table 2:** Measuring the stress, modulus, and strain that form during electro-chemical process of the various electrode materials for Li-ion batteries with the MOSS.

Material	Parameter			Ref.
	Stress (GPa)	Modulus (GPa)	Strain (%)	
Si	1.75	113	1.35	[67,73]
Li_0.32_Si		70		[66]
Li_0.47_Si	0.8	58.5	1.75	[73]
Li_1.4_Si	0.54	49.6	1.85	[73]
Li_2.34_Si	0.46	42.9	1.91	[73]
Li_3.0_Si		35		[66]
Li_3.5_Si	0.5			[72]
Graphite	0.25			[70]
Li_1.2_Ni_0.15_Mn_0.55_Co_0.1_O_2_	0.006			[69]
Battery casing (cylindrical spirally wound configuration)	0.001			[69]

**Table 3:** Measuring the stress, modulus, and strain that form during electro-chemical processes of the various electrode materials for Li-ion batteries with the DIC.

Material	Battery State	Parameter			Ref.
		Stress (GPa)	Strain (%)	Modulus (GPa)	
**Composite graphite electrodes**	1 cycle Cu	0.253	0.35	16.7	[81]
	55 cycle Cu	0.22	0.27	10.6	
**SiO@C composite electrodes**	Lithiation phase 100% SOC	−0.01189	3.00	0.12	[84]
**Graphite electrode**	75% lithiation	−0.529	0.178	0.244	[85]
	100% lithiation	−7.08	0.203	0.272	
**V_2_O_5_**	100% SOC	0.0282	1.3	0.61858	[83]

**Table 4:** Measuring the stress, partial molar volume, modulus, and strain that form during electro-chemical processes of the various electrode materials for Li-ion batteries with the BCMS.

Materials	Battery State	Stress (GPa)	Partial Molar Volume (10^−6^)	Strain (10^−3^)	Modulus (GPa)	References
Si	17% SOC (lithiation)	−0.0112	8.2	0.11	0.1	[103]
	17% SOC (delithiation)	−0.0098	0.5	0.005	0.3	
Graphite composite electrodes	40% SOC		1.88	0.007	31.9	[96]
Silicon carbide composite electrodes	40% SOC (lithiation)	−0.0148		0.618	0.38	[104]
Composite silicon electrode	30% SOC (lithiation)	−0.015			0.18	[30]

## Data Availability

No new data were created.

## References

[1] Dresselhaus M.S., Thomas I. (2001). Alternative energy technologies. Nature.

[2] Mukhopadhyay A., Sheldon B.W. (2014). Deformation and stress in electrode materials for Li-ion batteries. Prog. Mater. Sci..

[3] Kang K., Meng Y.S., Breger J., Grey C.P., Ceder G. (2006). Electrodes with high power and high capacity for rechargeable lithium batteries. Science.

[4] Tarascon J.-M., Armand M. (2001). Issues and challenges facing rechargeable lithium batteries. Nature.

[5] Wachsman E.D., Lee K.T. (2011). Lowering the temperature of solid oxide fuel cells. Science.

[6] Wang Y., Zhang Q., Li D., Hu J., Xu J., Dang D., Xiao X., Cheng Y.T. (2018). Mechanical property evolution of silicon composite electrodes studied by environmental nanoindentation. Adv. Energy Mater..

[7] Cui Z.W., Gao F., Qu J.M. (2012). A finite deformation stress-dependent chemical potential and its applications to lithium ion batteries. J. Mech. Phys. Solids.

[8] Bower A.F., Guduru P.R., Sethuraman V.A. (2011). A finite strain model of stress, diffusion, plastic flow, and electrochemical reactions in a lithium-ion half-cell. J. Mech. Phys. Solids.

[9] Harris S.J., Timmons A., Baker D.R., Monroe C. (2010). Direct in situ measurements of Li transport in Li-ion battery negative electrodes. Chem. Phys. Lett..

[10] He Y.-L., Hu H., Song Y.-C., Guo Z.-S., Liu C., Zhang J.-Q. (2014). Effects of concentration-dependent elastic modulus on the diffusion of lithium ions and diffusion induced stress in layered battery electrodes. J. Power Sources.

[11] Xiong R., Pan Y., Shen W., Li H., Sun F. (2020). Lithium-ion battery aging mechanisms and diagnosis method for automotive applications: Recent advances and perspectives. Renew. Sustain. Energy Rev..

[12] Vykhodtsev A.V., Jang D., Wang Q., Rosehart W., Zareipour H. (2022). A review of modelling approaches to characterize lithium-ion battery energy storage systems in techno-economic analyses of power systems. Renew. Sustain. Energy Rev..

[13] Yang Y., Zhou Q., Zhang L., Du D., Zheng M., Niu Q., Gao L., Yuan X. (2022). Recent progresses in state estimation of lithium-ion battery energy storage systems: A review. Trans. Inst. Meas. Control.

[14] Zhao K.J., Pharr M., Vlassak J.J., Suo Z.G. (2010). Fracture of electrodes in lithium-ion batteries caused by fast charging. J. Appl. Phys..

[15] Li A.G., West A.C., Preindl M. (2022). Towards unified machine learning characterization of lithium-ion battery degradation across multiple levels: A critical review. Appl. Energy.

[16] O’Kane S.E., Ai W., Madabattula G., Alonso-Alvarez D., Timms R., Sulzer V., Edge J.S., Wu B., Offer G.J., Marinescu M. (2022). Lithium-ion battery degradation: How to model it. Phys. Chem. Chem. Phys..

[17] Zheng H., Tan L., Liu G., Song X., Battaglia V.S. (2012). Calendering effects on the physical and electrochemical properties of Li Ni_1/3_Mn_1/3_Co_1/3_ O_2_ cathode. J. Power Sources.

[18] Song J., Han X., Gaskell K.J., Xu K., Lee S.B., Hu L. (2014). Enhanced electrochemical stability of high-voltage LiNi_0.5_Mn_1.5_O_4_ cathode by surface modification using atomic layer deposition. J. Nanopart. Res..

[19] Li J., Yang M., Zhang X., Wen J., Wang C., Huang G., Song W. (2023). First-Principles Study of the Effect of Ni-Doped on the Spinel-Type Mn-Based Cathode Discharge. ACS Appl. Mater. Interfaces.

[20] Sim R., Lee S., Li W., Manthiram A. (2021). Influence of Calendering on the Electrochemical Performance of LiNi_0.9_Mn_0.05_Al_0.05_O_2_ Cathodes in Lithium-Ion Cells. ACS Appl. Mater. Interfaces.

[21] Stoney G.G. (1909). The tension of metallic films deposited by electrolysis. Proc. R. Soc. Lond. Ser. A Contain. Pap. A Math. Phys. Character.

[22] Freund L.B., Suresh S. (2004). Thin Film Materials: Stress, Defect Formation and Surface Evolution.

[23] Sethuraman V.A., Nguyen A., Chon M.J., Nadimpalli S.P., Wang H., Abraham D.P., Bower A.F., Shenoy V.B., Guduru P.R. (2013). Stress evolution in composite silicon electrodes during lithiation/delithiation. J. Electrochem. Soc..

[24] Li H., Zhu C., Xu G., Luo L. (2016). Experimental identification of thermal induced warpage in polymer–metal composite films. Microelectron. Reliab..

[25] Pan B., Wu D., Xia Y. (2012). An active imaging digital image correlation method for deformation measurement insensitive to ambient light. Opt. Laser Technol..

[26] Song H., Liu C., Zhang H., Yang X., Chen Y., Leen S.B. (2021). Experimental investigation on damage evolution in pre-corroded aluminum alloy 7075-T7651 under fatigue loading. Mater. Sci. Eng. A.

[27] Jangid M.K., Mukhopadhyay A. (2019). Real-time monitoring of stress development during electrochemical cycling of electrode materials for Li-ion batteries: Overview and perspectives. J. Mater. Chem. A.

[28] Jones E., Silberstein M., White S.R., Sottos N.R. (2014). In situ measurements of strains in composite battery electrodes during electrochemical cycling. Exp. Mech..

[29] Chen J., Thapa A.K., Berfield T.A. (2014). In-situ characterization of strain in lithium battery working electrodes. J. Power Sources.

[30] Li D., Wang Y., Hu J., Lu B., Cheng Y.-T., Zhang J. (2017). In situ measurement of mechanical property and stress evolution in a composite silicon electrode. J. Power Sources.

[31] Li D., Wang Y. (2020). In-situ measurements of mechanical property and stress evolution of commercial graphite electrode. Mater. Des..

[32] Xie H., Kang Y., Song H., Guo J., Zhang Q. (2020). In situ method for stress measurements in film-substrate electrodes during electrochemical processes: Key role of softening and stiffening. Acta Mech. Sin..

[33] Guo Z., Liu C., Lu B., Feng J. (2019). Theoretical and experimental study on the interfacial adhesive properties of graphite electrodes in different charging and aging states. Carbon.

[34] Huang P., Liu C., Guo Z., Feng J. (2021). Analytical model and experimental verification of the interfacial peeling strength of electrodes. Exp. Mech..

[35] Yang S.-z., Huang Y.-f., Han X.-c., Han G.-h. (2021). Enhancing electrochemical performance of SnO2 anode with humic acid modification. Trans. Nonferrous Met. Soc. China.

[36] S Bhattacharyya A., P Kumar R., Acharya G., Ranjan V. (2017). Nanoindentation and Scratch test on Thin Film Energy Materials. Curr. Smart Mater..

[37] Bessette S., Hovington P., Demers H., Golozar M., Bouchard P., Gauvin R., Zaghib K. (2019). In-situ characterization of lithium native passivation layer in a high vacuum scanning electron microscope. Microsc. Microanal..

[38] Dai C., Zhu X., Pan J., Liao X., Pan Y. (2019). Mechanical Abuse Simulation and Effect of Graphene Oxides on Thermal Runaway of Lithium ion Batteries. Int. J. Electrochem. Sci..

[39] Gabbardo A.D., Frankel G. (2020). Hydrogen evolution on bare Mg surfaces using the scratched electrode technique. Corros. Sci..

[40] Sharifi H., Aliofkhazraei M., Darband G.B., Shrestha S. (2018). A review on adhesion strength of peo coatings by scratch test method. Surf. Rev. Lett..

[41] Hovington P., Dontigny M., Guerfi A., Trottier J., Lagacé M., Mauger A., Julien C., Zaghib K. (2014). In situ Scanning electron microscope study and microstructural evolution of nano silicon anode for high energy Li-ion batteries. J. Power Sources.

[42] Tsuda T., Kanetsuku T., Sano T., Oshima Y., Ui K., Yamagata M., Ishikawa M., Kuwabata S. (2015). In situ SEM observation of the Si negative electrode reaction in an ionic-liquid-based lithium-ion secondary battery. Microscopy.

[43] Gómez-Cámer J.L., Bünzli C., Hantel M.M., Poux T., Novák P. (2016). On the correlation between electrode expansion and cycling stability of graphite/Si electrodes for Li-ion batteries. Carbon.

[44] Zhou X., Li T., Cui Y., Fu Y., Liu Y., Zhu L. (2019). In situ focused ion beam scanning electron microscope study of microstructural evolution of single tin particle anode for Li-ion batteries. ACS Appl. Mater. Interfaces.

[45] Chen C.-Y., Sano T., Tsuda T., Ui K., Oshima Y., Yamagata M., Ishikawa M., Haruta M., Doi T., Inaba M. (2016). In situ scanning electron microscopy of silicon anode reactions in lithium-ion batteries during charge/discharge processes. Sci. Rep..

[46] Shao M. (2014). In situ microscopic studies on the structural and chemical behaviors of lithium-ion battery materials. J Power Sources.

[47] Harris S.J., Rahani E.K., Shenoy V.B. (2012). Direct in situ observation and numerical simulations of non-shrinking-core behavior in an MCMB graphite composite electrode. J. Electrochem. Soc..

[48] Yang L., Chen H.-S., Jiang H., Wei Y.-J., Song W.-L., Fang D.-N. (2018). Failure mechanisms of 2D silicon film anodes: In situ observations and simulations on crack evolution. Chem. Commun..

[49] Wang X., Zeng W., Hong L., Xu W., Yang H., Wang F., Duan H., Tang M., Jiang H. (2018). Stress-driven lithium dendrite growth mechanism and dendrite mitigation by electroplating on soft substrates. Nat. Energy.

[50] Li N., Xin Y., Chen H., Jiao S., Jiang H., Song W.-L., Fang D. (2019). Thickness evolution of graphite-based cathodes in the dual ion batteries via in operando optical observation. J. Energy Chem..

[51] Breitung B., Baumann P., Sommer H., Janek J., Brezesinski T. (2016). In situ and operando atomic force microscopy of high-capacity nano-silicon based electrodes for lithium-ion batteries. Nanoscale.

[52] Yoon I., Abraham D.P., Lucht B.L., Bower A.F., Guduru P.R. (2016). In situ measurement of solid electrolyte interphase evolution on silicon anodes using atomic force microscopy. Adv. Energy Mater..

[53] Mahankali K., Thangavel N.K., Reddy Arava L.M. (2019). In situ electrochemical mapping of lithium–sulfur battery interfaces using AFM–SECM. Nano Lett..

[54] Wang J., Zhang X., Li Z., Ma Y., Ma L. (2020). Recent progress of biomass-derived carbon materials for supercapacitors. J. Power Sources.

[55] Hu J., Wang Y., Li D., Cheng Y.-T. (2018). Effects of adhesion and cohesion on the electrochemical performance and durability of silicon composite electrodes. J. Power Sources.

[56] Zou R., Cui Z., Liu Q., Guan G., Zhang W., He G., Yang J., Hu J. (2017). In situ transmission electron microscopy study of individual nanostructures during lithiation and delithiation processes. J. Mater. Chem. A.

[57] Wheatcroft L., Özkaya D., Cookson J., Inkson B.J. (2018). Towards in-situ TEM for Li-ion Battery Research. Energy Procedia.

[58] Xie H., Tan X., Luber E.J., Olsen B.C., Kalisvaart W.P., Jungjohann K.L., Mitlin D., Buriak J.M. (2018). β-SnSb for sodium ion battery anodes: Phase transformations responsible for enhanced cycling stability revealed by in situ TEM. ACS Energy Lett..

[59] Ji Y.-R., Weng S.-T., Li X.-Y., Zhang Q.-H., Gu L. (2020). Atomic-scale structural evolution of electrode materials in Li-ion batteries: A review. Rare Met..

[60] Zheng H., Lu X., He K. (2022). In situ transmission electron microscopy and artificial intelligence enabled data analytics for energy materials. J. Energy Chem..

[61] Sun Z., Pan J., Chen W., Chen H., Zhou S., Wu X., Wang Y., Kim K., Li J., Liu H. (2024). Electrochemical Processes and Reactions In Rechargeable Battery Materials Revealed via In Situ Transmission Electron Microscopy. Adv. Energy Mater..

[62] Cui J., Zheng H., He K. (2021). In situ TEM study on conversion-type electrodes for rechargeable ion batteries. Adv. Mater..

[63] Sethuraman V.A., Srinivasan V., Bower A.F., Guduru P.R. (2010). In Situ Measurements of Stress-Potential Coupling in Lithiated Silicon. J. Electrochem. Soc..

[64] Brantley W.A. (1973). Calculated elastic constants for stress problems associated with semiconductor devices. J. Appl. Phys..

[65] Beaulieu L.Y., Hatchard T.D., Bonakdarpour A., Fleischauer M.D., Dahn J.R. (2003). Reaction of Li with alloy thin films studied by in situ AFM. J. Electrochem. Soc..

[66] Sethuraman V.A., Chon M.J., Shimshak M., Van Winkle N., Guduru P.R. (2010). In situ measurement of biaxial modulus of Si anode for Li-ion batteries. Electrochem. Commun..

[67] Sethuraman V.A., Chon M.J., Shimshak M., Srinivasan V., Guduru P.R. (2010). In situ measurements of stress evolution in silicon thin films during electrochemical lithiation and delithiation. J. Power Sources.

[68] Sheth J., Karan N.K., Abraham D.P., Nguyen C.C., Lucht B.L., Sheldon B.W., Guduru P.R. (2016). In situ stress evolution in Li1+ xMn2O4 thin films during electrochemical cycling in li-ion cells. J. Electrochem. Soc..

[69] Nadimpalli S.P.V., Sethuraman V.A., Abraham D.P., Bower A.F., Guduru P.R. (2015). Stress Evolution in Lithium-Ion Composite Electrodes during Electrochemical Cycling and Resulting Internal Pressures on the Cell Casing. J. Electrochem. Soc..

[70] Mukhopadhyay A., Tokranov A., Sena K., Xiao X., Sheldon B.W. (2011). Thin film graphite electrodes with low stress generation during Li-intercalation. Carbon.

[71] Sethuraman V.A., Van Winkle N., Abraham D.P., Bower A.F., Guduru P.R. (2012). Real-time stress measurements in lithium-ion battery negative-electrodes. J. Power Sources.

[72] Chon M.J., Sethuraman V.A., McCormick A., Srinivasan V., Guduru P.R. (2011). Real-time measurement of stress and damage evolution during initial lithiation of crystalline silicon. Phys. Rev. Lett..

[73] Choi Y.S., Pharr M., Oh K.H., Vlassak J.J. (2015). A simple technique for measuring the fracture energy of lithiated thin-film silicon electrodes at various lithium concentrations. J. Power Sources.

[74] Nation L., Li J., James C., Qi Y., Dudney N., Sheldon B.W. (2017). In situ stress measurements during electrochemical cycling of lithium-rich cathodes. J. Power Sources.

[75] Koga H., Croguennec L., Ménétrier M., Mannessiez P., Weill F., Delmas C. (2013). Different oxygen redox participation for bulk and surface: A possible global explanation for the cycling mechanism of Li_1.20_Mn_0.54_CO_0.13_Ni_0.13_O_2_. J. Power Sources.

[76] Lee E., Persson K.A. (2014). Structural and Chemical Evolution of the Layered Li-Excess Li*_x_*MnO_3_ as a Function of Li Content from First-Principles Calculations. Adv. Energy Mater..

[77] Xie H., Yang W., Kang Y., Zhang Q., Han B., Qiu W. (2021). In-situ strain field measurement and mechano-electro-chemical analysis of graphite electrodes via fluorescence digital image correlation. Exp. Mech..

[78] Li C., Yang S., Xin L., Wang Z., Xu Q., Li L., Wang S. (2021). In-Situ Characterization for Solid Electrolyte Deformations in a Lithium Metal Solid-State Battery. J. Electrochem. Soc..

[79] Özdogru B., Dykes H., Gregory D., Saurel D., Murugesan V., Casas-Cabanas M., Çapraz Ö.Ö. (2021). Elucidating cycling rate-dependent electrochemical strains in sodium iron phosphate cathodes for Na-ion batteries. J. Power Sources.

[80] Tao R., Zhu J., Zhang Y., Song W.-L., Chen H., Fang D. (2020). Quantifying the 2D anisotropic displacement and strain fields in graphite-based electrode via in situ scanning electron microscopy and digital image correlation. Extrem. Mech. Lett..

[81] Luo J., Dai C., Wang Z., Liu K., Mao W., Fang D., Chen X. (2016). In-situ measurements of mechanical and volume change of LiCoO_2_ lithium-ion batteries during repeated charge–discharge cycling by using digital image correlation. Measurement.

[82] Yang W., Xie H., Shi B., Song H., Qiu W., Zhang Q. (2019). In-situ experimental measurements of lithium concentration distribution and strain field of graphite electrodes during electrochemical process. J. Power Sources.

[83] Mao W., Wang Z., Li C., Zhu X., Dai C., Yang H., Chen X., Fang D. (2018). In-situ characterizations of chemo-mechanical behavior of free-standing vanadium pentoxide cathode for lithium-ion batteries during discharge-charge cycling using digital image correlation. J. Power Sources.

[84] Dai C., Li C., Huang H., Wang Z., Zhu X., Liao X., Chen X., Pan Y., Fang D. (2019). In situ strain measurements and stress analysis of SiO@ C composite electrodes during electrochemical cycling by using digital image correlation. Solid State Ion..

[85] Xie H., Han B., Song H., Li X., Kang Y., Zhang Q. (2021). In-situ measurements of electrochemical stress/strain fields and stress analysis during an electrochemical process. J. Mech. Phys. Solids.

[86] Mukaibo H., Momma T., Shacham-Diamand Y., Osaka T., Kodaira M. (2007). In situ stress transition observations of electrodeposited Sn-based anode materials for lithium-ion secondary batteries. Electrochem. Solid-State Lett..

[87] Xie H., Qiu W., Song H., Tian J. (2016). In situ measurement of the deformation and elastic modulus evolution in Si composite electrodes during electrochemical lithiation and delithiation. J. Electrochem. Soc..

[88] Xie H., Zhang Q., Song H., Shi B., Kang Y. (2017). Modeling and in situ characterization of lithiation-induced stress in electrodes during the coupled mechano-electro-chemical process. J. Power Sources.

[89] Tavassol H., Jones E.M., Sottos N.R., Gewirth A.A. (2016). Electrochemical stiffness in lithium-ion batteries. Nat. Mater..

[90] Özdogru B., Dykes H., Padwal S., Harimkar S., Çapraz Ö.Ö. (2020). Electrochemical strain evolution in iron phosphate composite cathodes during lithium and sodium ion intercalation. Electrochim. Acta.

[91] Mao W., Zhu X., Zhang Z., Huang H., Dai C., Pan J., Pan Y., Chen X., Fang D. (2020). Measurements of fracture properties of MWCNTs modified LiNi_0.5_Mn_0.3_Co_0.2_O_2_ electrodes by a modified shear lag model. Mater. Sci. Eng. A.

[92] Zheng H., Zhang L., Liu G., Song X., Battaglia V.S. (2012). Correlationship between electrode mechanics and long-term cycling performance for graphite anode in lithium ion cells. J. Power Sources.

[93] Wu Z., Yang X., Sheng K., Li D. (2024). Experimental Investigation and Controllability Study of Electrochemical Actuators Based on Si/CNTs Composite Material. J. Electrochem. Energy Convers. Storage.

[94] Zhang J., Lu B., Song Y., Ji X. (2012). Diffusion induced stress in layered Li-ion battery electrode plates. J. Power Sources.

[95] Yu H., Li J., Jiang H., Li W., Li G., Li D. (2023). Chemo-Mechanical Coupling Measurement of LiMn_2_O_4_ Composite Electrode during Electrochemical Cycling. Batteries.

[96] Li D., Wang Y., Lu B., Zhang J. (2020). Real-time measurements of electro-mechanical coupled deformation and mechanical properties of commercial graphite electrodes. Carbon.

[97] Li D., Li Z., Song Y., Zhang J. (2016). Analysis of diffusion induced elastoplastic bending of bilayer lithium-ion battery electrodes. Appl. Math. Mech..

[98] Li D., Wang Y., Hu J., Lu B., Dang D., Zhang J., Cheng Y.-T. (2018). Role of polymeric binders on mechanical behavior and cracking resistance of silicon composite electrodes during electrochemical cycling. J. Power Sources.

[99] Li D., Liu H., Wan H., Wang Y., Zhang J. (2022). Real time characterization of the current collector’s role on the electro-chemo-mechanical coupling performance of Si composite electrode. J. Energy Storage.

[100] Li D., Zhu G., Liu H., Wang Y. (2022). Diffusion-induced stress in commercial graphite electrodes during multiple cycles measured by an in situ method. Micromachines.

[101] Li D., Wang Y. (2020). Communication—Controllable Deformation of Composite Graphite Electrodes during Electrochemical Process. J. Electrochem. Soc..

[102] Liu H., Zhang G., Li D., Zhang J. (2022). An Improved Experiment for Measuring Lithium Concentration-Dependent Material Properties of Graphite Composite Electrodes. Nanomaterials.

[103] Li D., Wan H., Liu H., Wang Y., Zhang J. (2022). Experimental measurement of electro-chemo-mechanical properties of a composite silicon electrode in lithium ion batteries. Phys. Chem. Chem. Phys..

[104] Yu H., Liu X., Li D. (2022). Experimental measurement of stress evolution in silicon carbide composite electrode during electrochemical cycling. Mater. Sci. Semicond. Process..

